# The Necessity of Re-vaccination

**Published:** 1854-02

**Authors:** 


### THE NECESSITY OF RE-VACCINATION.

Dr. G. Benedict, physician to the New York Northwestern Dispensary, closes an article on this subject, in the *New York Journal of Medicine*, with the following summary, the result of his experience in vaccinia as a preventive of small pox.

"That vaccination, properly performed, and repeated until the susceptibility to the vaccine disease is exhausted from the system affords entire immunity from the variolous disease.

"It may seem that, by including so much, my proposition is worthless, as it would extinguish not only the genuine disease, but its modification, varioloid. But we are to bear in mind that one two or three successive pustules may still leave the system unprotected, at least in part. Vaccination should be repeated until nothing like a pustule can be obtained. Let each one observe for himself, until evidence accumulates which shall sustain or overthrow this position; and let no one say that vaccination is not a protection for those in whom the susceptibility to variola is unusually strong, until he first ascertains whether there is not still left some susceptibility to vaccinia."

